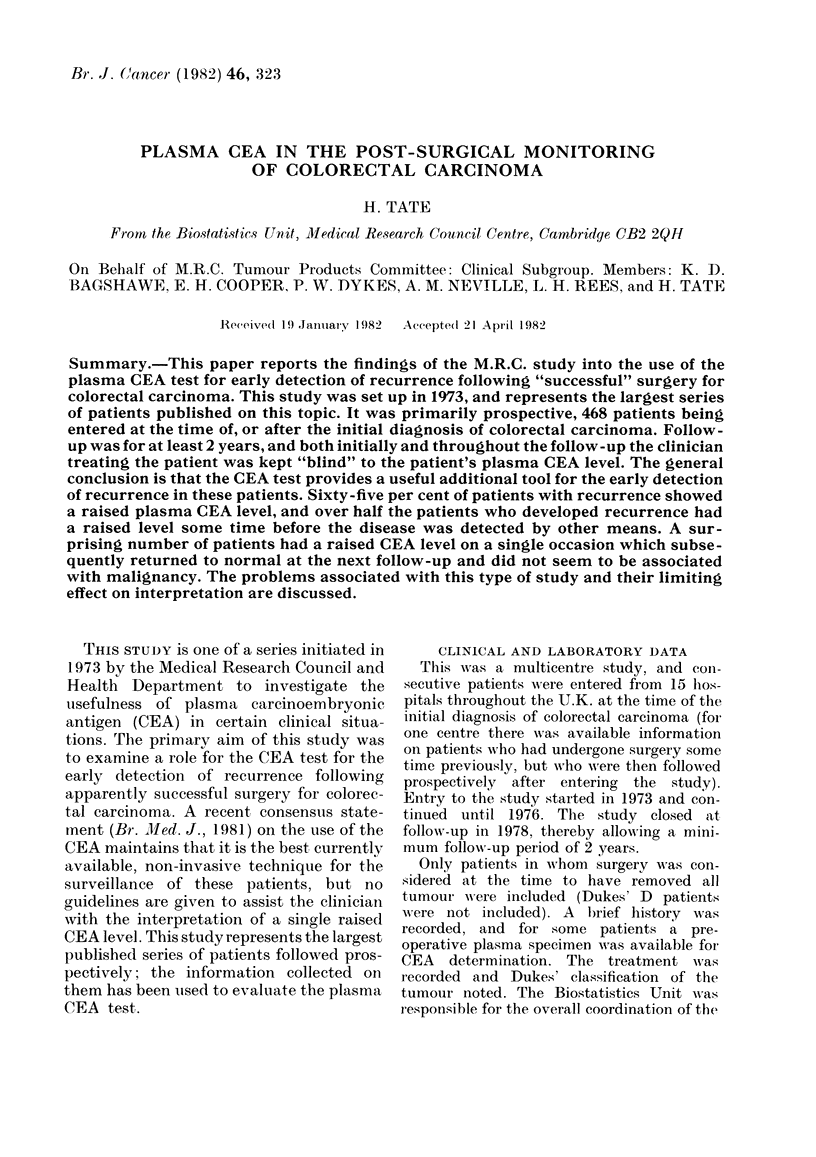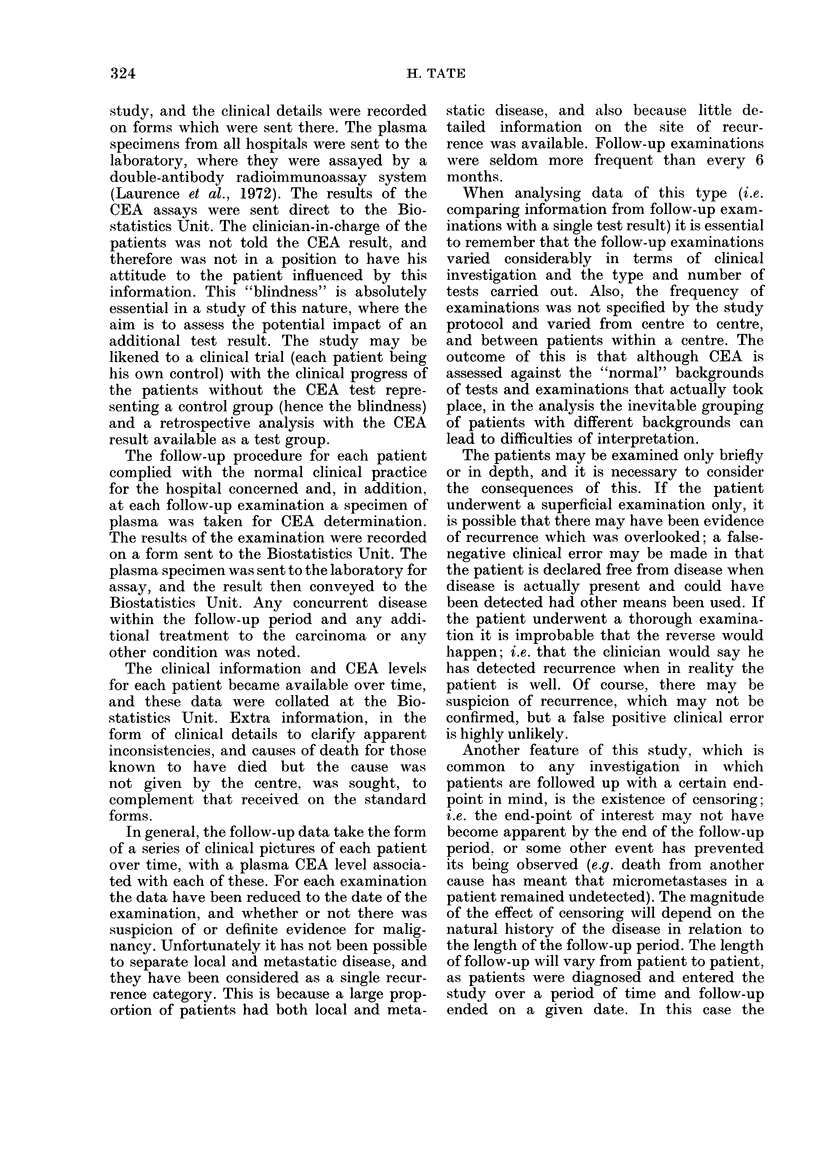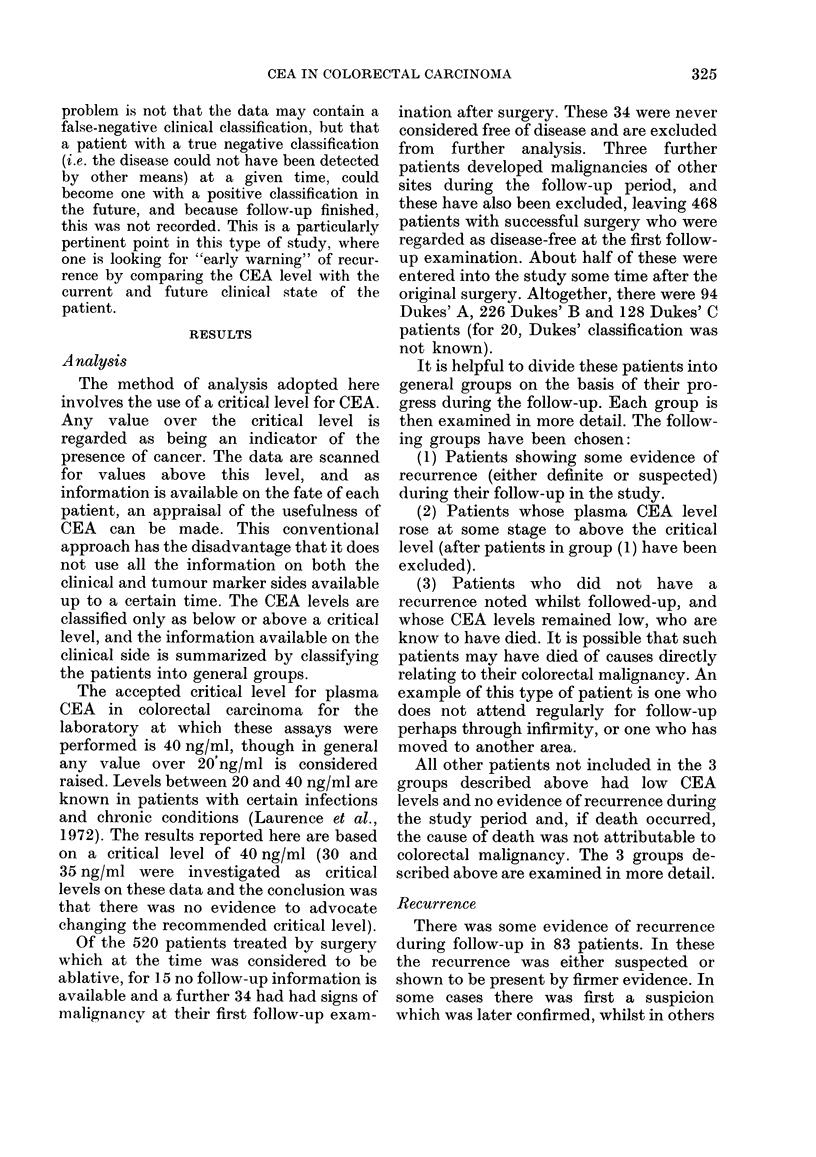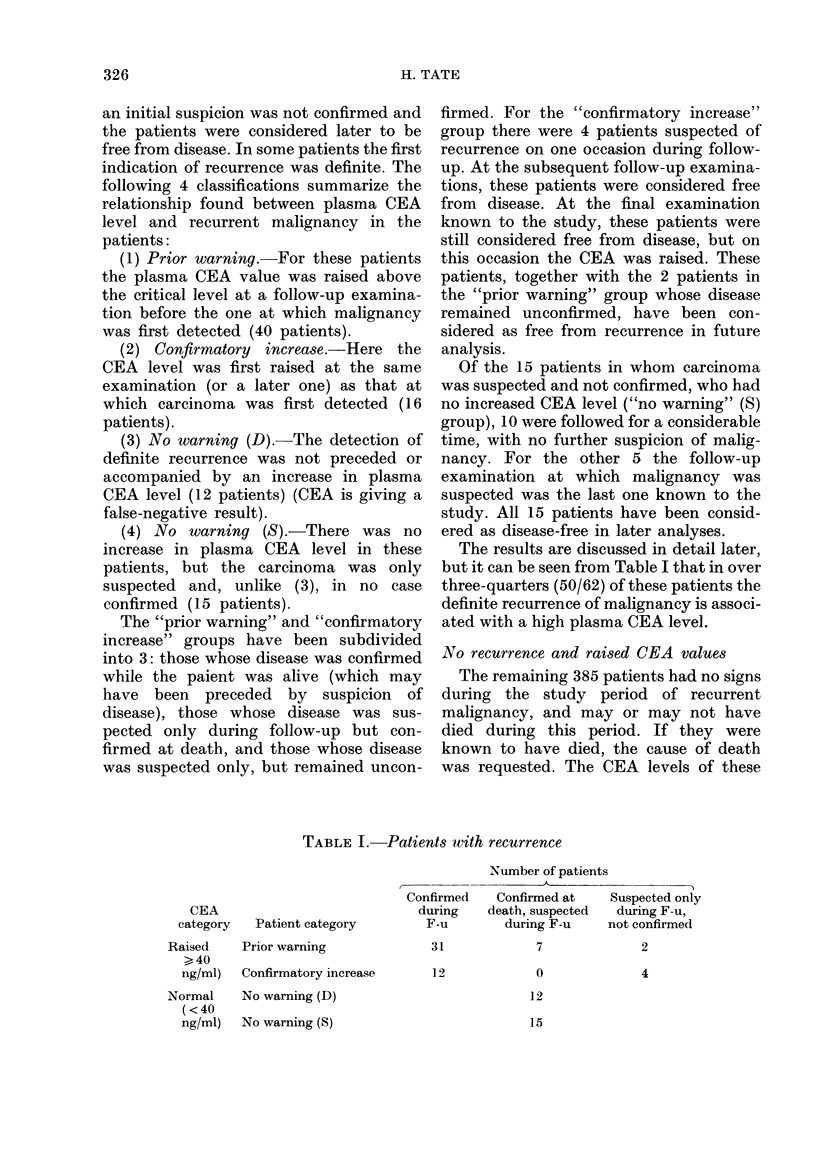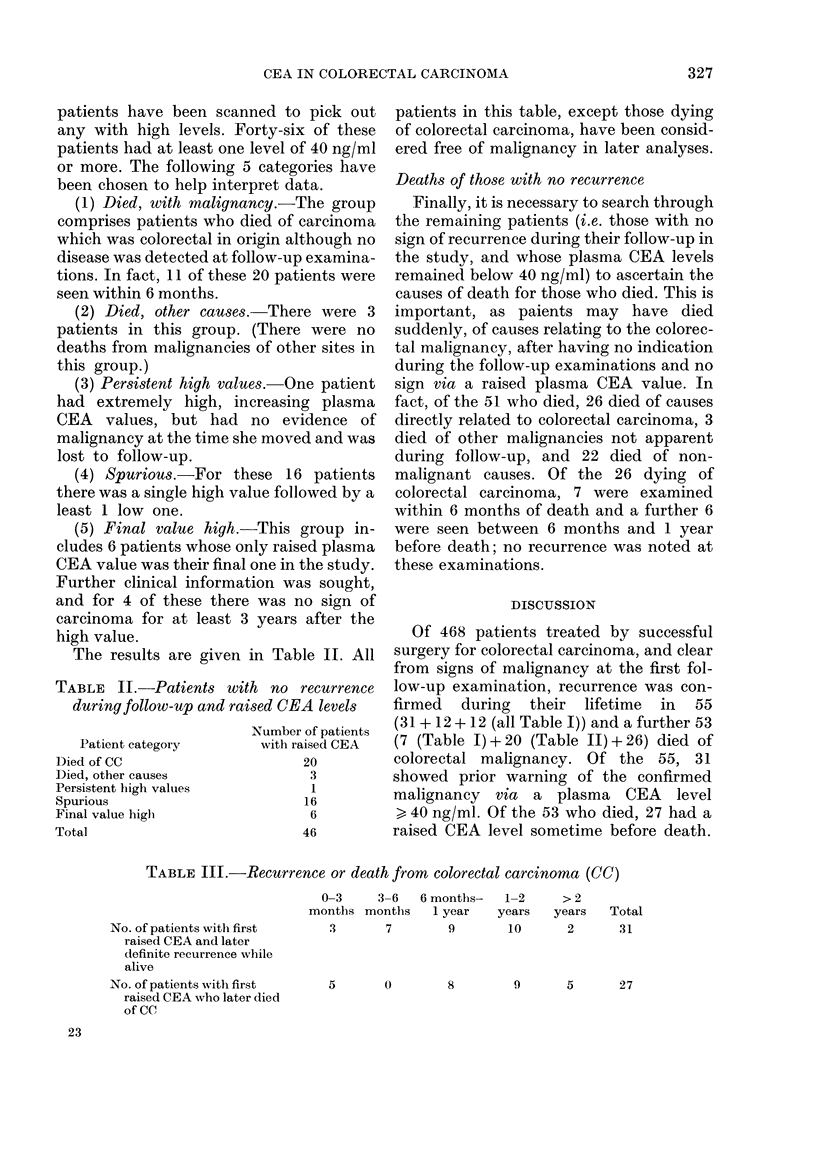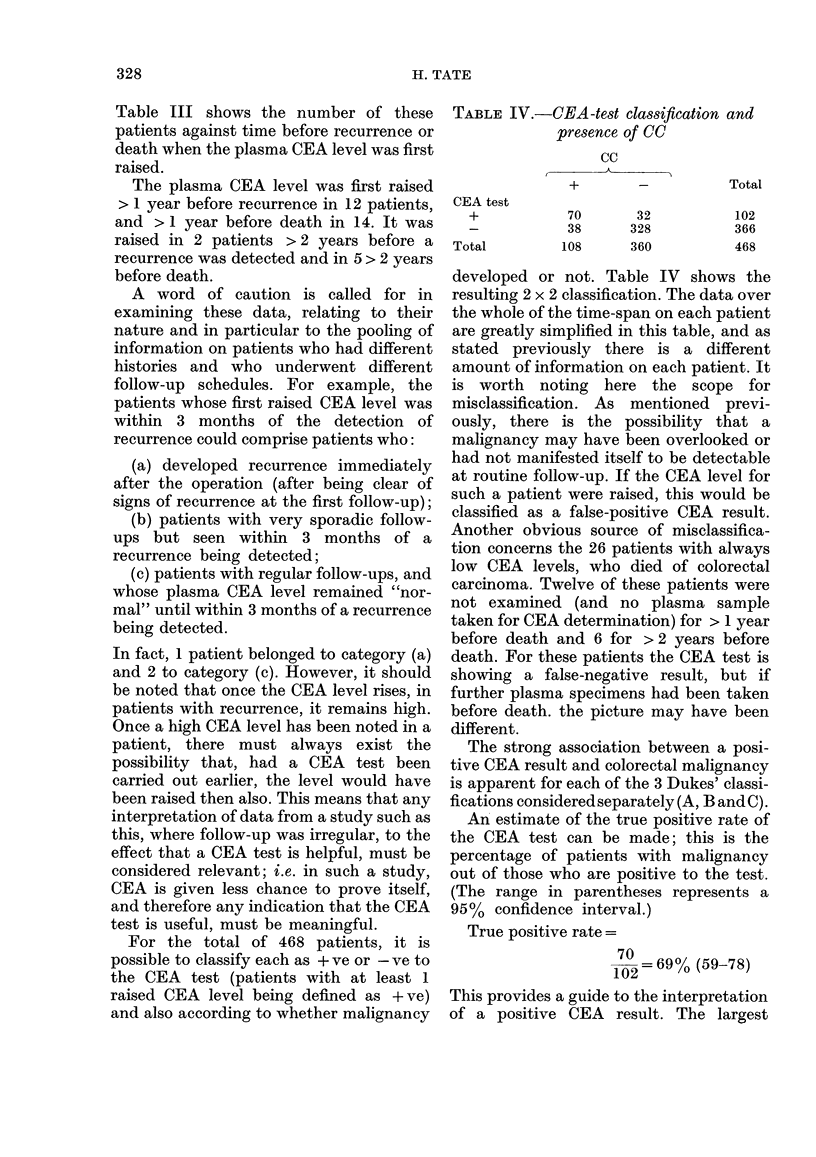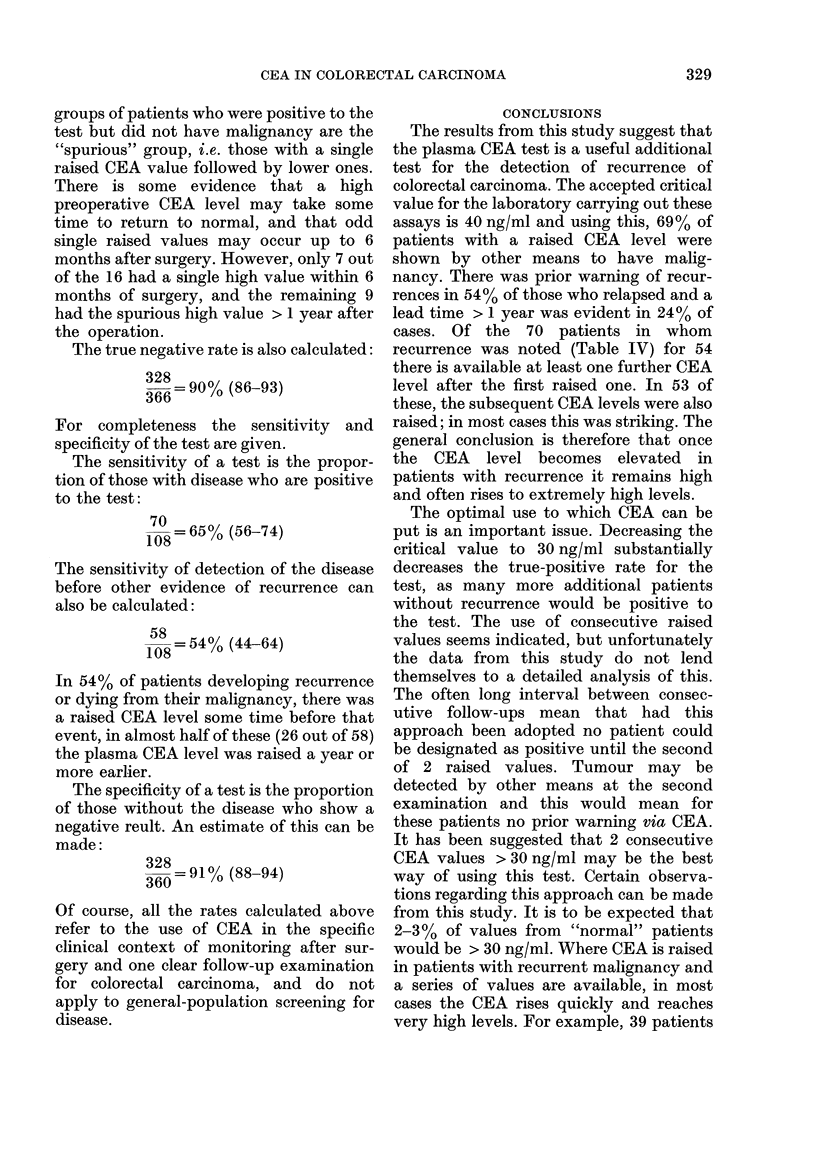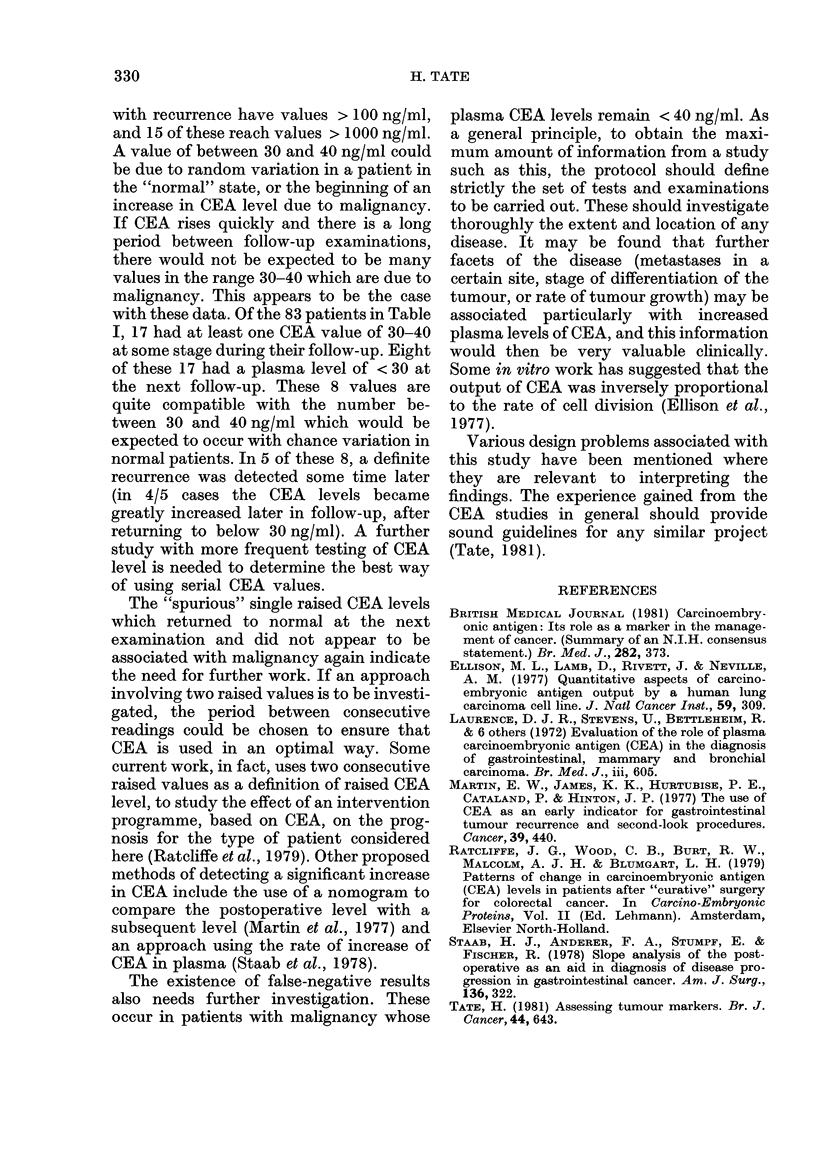# Plasma CEA in the post-surgical monitoring of colorectal carcinoma.

**DOI:** 10.1038/bjc.1982.207

**Published:** 1982-09

**Authors:** H. Tate

## Abstract

This paper reports the findings of the M.R.C., study into the use of the plasma CEA test for early detection of recurrence following "successful" surgery for colorectal carcinoma. This study was set up in 1973, and represents the largest series of patients published on this topic. It was primarily prospective, 468 patients being entered at the time of, or after the initial diagnosis of colorectal carcinoma. Follow-up was for at least 2 years, and both initially and throughout the follow-up the clinician treating the patient was kept "blind" to the patient's plasma CEA level. The general conclusion is that the CEA test provides a useful additional tool for the early detection of recurrence in these patients. Sixty-five per cent of patients with recurrence showed a raised plasma CEA level, and over half the patients who developed recurrence had a raised level some time before the disease was detected by other means. A surprising number of patients had a raised CEA level on a single occasion which subsequently returned to normal at the next follow-up and did not seem to be associated with malignancy. The problems associated with this type of study and their limiting effect on interpretation are discussed.


*Br. J. Cancer* (1982) **46,** 323

### PLASMA CEA IN THE POST-SURGICAL MONITORING OF COLORECTAL CARCINOMA

H. TATE

*From the Biostatistics Unit, Medical Research Council Centre, Cambridge CB2 2QH*

On Behalf of M.R.C. Tumour Products Committee: Clinical Subgroup. Members: K. D. BAGSHAWE, E. H. COOPER, P. W. DYKES, A. M. NEVILLE, L. H. REES, and H. TATE

Received 19 January 1982 Accepted 21 April 1982

**Summary.—This paper reports the findings of the M.R.C. study into the use of the plasma CEA test for early detection of recurrence following "successful" surgery for colorectal carcinoma. This study was set up in 1973, and represents the largest series of patients published on this topic. It was primarily prospective, 468 patients being entered at the time of, or after the initial diagnosis of colorectal carcinoma. Follow-up was for at least 2 years, and both initially and throughout the follow-up the clinician treating the patient was kept "blind" to the patient's plasma CEA level. The general conclusion is that the CEA test provides a useful additional tool for the early detection of recurrence in these patients. Sixty-five per cent of patients with recurrence showed a raised plasma CEA level, and over half the patients who developed recurrence had a raised level some time before the disease was detected by other means. A surprising number of patients had a raised CEA level on a single occasion which subsequently returned to normal at the next follow-up and did not seem to be associated with malignancy. The problems associated with this type of study and their limiting effect on interpretation are discussed.**

THIS STUDY is one of a series initiated in 1973 by the Medical Research Council and Health Department to investigate the usefulness of plasma carcinoembryonic antigen (CEA) in certain clinical situations. The primary aim of this study was to examine a role for the CEA test for the early detection of recurrence following apparently successful surgery for colorectal carcinoma. A recent consensus statement (*Br. Med. J.*, 1981) on the use of the CEA maintains that it is the best currently available, non-invasive technique for the surveillance of these patients, but no guidelines are given to assist the clinician with the interpretation of a single raised CEA level. This study represents the largest published series of patients followed prospectively; the information collected on them has been used to evaluate the plasma CEA test.

### CLINICAL AND LABORATORY DATA

This was a multicentre study, and consecutive patients were entered from 15 hospitals throughout the U.K. at the time of the initial diagnosis of colorectal carcinoma (for one centre there was available information on patients who had undergone surgery some time previously, but who were then followed prospectively after entering the study). Entry to the study started in 1973 and continued until 1976. The study closed at follow-up in 1978, thereby allowing a minimum follow-up period of 2 years.

Only patients in whom surgery was considered at the time to have removed all tumour were included (Dukes' D patients were not included). A brief history was recorded, and for some patients a preoperative plasma specimen was available for CEA determination. The treatment was recorded and Dukes' classification of the tumour noted. The Biostatistics Unit was responsible for the overall coordination of the study, and the clinical details were recorded on forms which were sent there. The plasma specimens from all hospitals were sent to the laboratory, where they were assayed by a double-antibody radioimmunoassay system (Laurence *et al.*, 1972). The results of the CEA assays were sent direct to the Biostatistics Unit. The clinician-in-charge of the patients was not told the CEA result, and therefore was not in a position to have his attitude to the patient influenced by this information. This "blindness" is absolutely essential in a study of this nature, where the aim is to assess the potential impact of an additional test result. The study may be likened to a clinical trial (each patient being his own control) with the clinical progress of the patients without the CEA test representing a control group (hence the blindness) and a retrospective analysis with the CEA result available as a test group.

The follow-up procedure for each patient complied with the normal clinical practice for the hospital concerned and, in addition, at each follow-up examination a specimen of plasma was taken for CEA determination. The results of the examination were recorded on a form sent to the Biostatistics Unit. The plasma specimen was sent to the laboratory for assay, and the result then conveyed to the Biostatistics Unit. Any concurrent disease within the follow-up period and any additional treatment to the carcinoma or any other condition was noted.

The clinical information and CEA levels for each patient became available over time, and these data were collated at the Biostatistics Unit. Extra information, in the form of clinical details to clarify apparent inconsistencies, and causes of death for those known to have died but the cause was not given by the centre, was sought, to complement that received on the standard forms.

In general, the follow-up data take the form of a series of clinical pictures of each patient over time, with a plasma CEA level associated with each of these. For each examination the data have been reduced to the date of the examination, and whether or not there was suspicion of or definite evidence for malignancy. Unfortunately it has not been possible to separate local and metastatic disease, and they have been considered as a single recurrence category. This is because a large proportion of patients had both local and metastatic disease, and also because little detailed information on the site of recurrence was available. Follow-up examinations were seldom more frequent than every 6 months.

When analysing data of this type (*i.e.* comparing information from follow-up examinations with a single test result) it is essential to remember that the follow-up examinations varied considerably in terms of clinical investigation and the type and number of tests carried out. Also, the frequency of examinations was not specified by the study protocol and varied from centre to centre, and between patients within a centre. The outcome of this is that although CEA is assessed against the "normal" backgrounds of tests and examinations that actually took place, in the analysis the inevitable grouping of patients with different backgrounds can lead to difficulties of interpretation.

The patients may be examined only briefly or in depth, and it is necessary to consider the consequences of this. If the patient underwent a superficial examination only, it is possible that there may have been evidence of recurrence which was overlooked; a false-negative clinical error may be made in that the patient is declared free from disease when disease is actually present and could have been detected had other means been used. If the patient underwent a thorough examination it is improbable that the reverse would happen; *i.e.* that the clinician would say he has detected recurrence when in reality the patient is well. Of course, there may be suspicion of recurrence, which may not be confirmed, but a false positive clinical error is highly unlikely.

Another feature of this study, which is common to any investigation in which patients are followed up with a certain end-point in mind, is the existence of censoring; *i.e.* the end-point of interest may not have become apparent by the end of the follow-up period, or some other event has prevented its being observed (*e.g.* death from another cause has meant that micrometastases in a patient remained undetected). The magnitude of the effect of censoring will depend on the natural history of the disease in relation to the length of the follow-up period. The length of follow-up will vary from patient to patient, as patients were diagnosed and entered the study over a period of time and follow-up ended on a given date. In this case the problem is not that the data may contain a false-negative clinical classification, but that a patient with a true negative classification (*i.e.* the disease could not have been detected by other means) at a given time, could become one with a positive classification in the future, and because follow-up finished, this was not recorded. This is a particularly pertinent point in this type of study, where one is looking for "early warning" of recurrence by comparing the CEA level with the current and future clinical state of the patient.

### RESULTS

### *Analysis*

The method of analysis adopted here involves the use of a critical level for CEA. Any value over the critical level is regarded as being an indicator of the presence of cancer. The data are scanned for values above this level, and as information is available on the fate of each patient, an appraisal of the usefulness of CEA can be made. This conventional approach has the disadvantage that it does not use all the information on both the clinical and tumour marker sides available up to a certain time. The CEA levels are classified only as below or above a critical level, and the information available on the clinical side is summarized by classifying the patients into general groups.

The accepted critical level for plasma CEA in colorectal carcinoma for the laboratory at which these assays were performed is 40 ng/ml, though in general any value over 20 ng/ml is considered raised. Levels between 20 and 40 ng/ml are known in patients with certain infections and chronic conditions (Laurence *et al.*, 1972). The results reported here are based on a critical level of 40 ng/ml (30 and 35 ng/ml were investigated as critical levels on these data and the conclusion was that there was no evidence to advocate changing the recommended critical level).

Of the 520 patients treated by surgery which at the time was considered to be ablative, for 15 no follow-up information is available and a further 34 had had signs of malignancy at their first follow-up examination after surgery. These 34 were never considered free of disease and are excluded from further analysis. Three further patients developed malignancies of other sites during the follow-up period, and these have also been excluded, leaving 468 patients with successful surgery who were regarded as disease-free at the first follow-up examination. About half of these were entered into the study some time after the original surgery. Altogether, there were 94 Dukes' A, 226 Dukes' B and 128 Dukes' C patients (for 20, Dukes' classification was not known).

It is helpful to divide these patients into general groups on the basis of their progress during the follow-up. Each group is then examined in more detail. The following groups have been chosen:

(1) Patients showing some evidence of recurrence (either definite or suspected) during their follow-up in the study.

(2) Patients whose plasma CEA level rose at some stage to above the critical level (after patients in group (1) have been excluded).

(3) Patients who did not have a recurrence noted whilst followed-up, and whose CEA levels remained low, who are know to have died. It is possible that such patients may have died of causes directly relating to their colorectal malignancy. An example of this type of patient is one who does not attend regularly for follow-up perhaps through infirmity, or one who has moved to another area.

All other patients not included in the 3 groups described above had low CEA levels and no evidence of recurrence during the study period and, if death occurred, the cause of death was not attributable to colorectal malignancy. The 3 groups described above are examined in more detail.

### *Recurrence*

There was some evidence of recurrence during follow-up in 83 patients. In these the recurrence was either suspected or shown to be present by firmer evidence. In some cases there was first a suspicion which was later confirmed, whilst in others an initial suspicion was not confirmed and the patients were considered later to be free from disease. In some patients the first indication of recurrence was definite. The following 4 classifications summarize the relationship found between plasma CEA level and recurrent malignancy in the patients:

(1) *Prior warning.*—For these patients the plasma CEA value was raised above the critical level at a follow-up examination before the one at which malignancy was first detected (40 patients).

(2) *Confirmatory increase.*—Here the CEA level was first raised at the same examination (or a later one) as that at which carcinoma was first detected (16 patients).

(3) *No warning (D).*—The detection of definite recurrence was not preceded or accompanied by an increase in plasma CEA level (12 patients) (CEA is giving a false-negative result).

(4) *No warning (S).*—There was no increase in plasma CEA level in these patients, but the carcinoma was only suspected and, unlike (3), in no case confirmed (15 patients).

The "prior warning" and "confirmatory increase" groups have been subdivided into 3: those whose disease was confirmed while the paient was alive (which may have been preceded by suspicion of disease), those whose disease was suspected only during follow-up but confirmed at death, and those whose disease was suspected only, but remained unconfirmed. For the "confirmatory increase" group there were 4 patients suspected of recurrence on one occasion during follow-up. At the subsequent follow-up examinations, these patients were considered free from disease. At the final examination known to the study, these patients were still considered free from disease, but on this occasion the CEA was raised. These patients, together with the 2 patients in the "prior warning" group whose disease remained unconfirmed, have been considered as free from recurrence in future analysis.

Of the 15 patients in whom carcinoma was suspected and not confirmed, who had no increased CEA level ("no warning" (S) group), 10 were followed for a considerable time, with no further suspicion of malignancy. For the other 5 the follow-up examination at which malignancy was suspected was the last one known to the study. All 15 patients have been considered as disease-free in later analyses.

The results are discussed in detail later, but it can be seen from Table I that in over three-quarters (50/62) of these patients the definite recurrence of malignancy is associated with a high plasma CEA level.

*No recurrence and raised CEA values*

The remaining 385 patients had no signs during the study period of recurrent malignancy, and may or may not have died during this period. If they were known to have died, the cause of death was requested. The CEA levels of these

TABLE I.—*Patients with recurrence*

| CEA category | Patient category | Number of patients: Confirmed during F-u | Confirmed at death, suspected during F-u | Suspected only during F-u, not confirmed |
|---|---|---|---|---|
| Raised ($\geqslant 40$ ng/ml) | Prior warning | 31 | 7 | 2 |
| | Confirmatory increase | 12 | 0 | 4 |
| Normal ($< 40$ ng/ml) | No warning (D) | | 12 | |
| | No warning (S) | | 15 | |

patients have been scanned to pick out any with high levels. Forty-six of these patients had at least one level of 40 ng/ml or more. The following 5 categories have been chosen to help interpret data.

(1) *Died, with malignancy.*—The group comprises patients who died of carcinoma which was colorectal in origin although no disease was detected at follow-up examinations. In fact, 11 of these 20 patients were seen within 6 months.

(2) *Died, other causes.*—There were 3 patients in this group. (There were no deaths from malignancies of other sites in this group.)

(3) *Persistent high values.*—One patient had extremely high, increasing plasma CEA values, but had no evidence of malignancy at the time she moved and was lost to follow-up.

(4) *Spurious.*—For these 16 patients there was a single high value followed by a least 1 low one.

(5) *Final value high.*—This group includes 6 patients whose only raised plasma CEA value was their final one in the study. Further clinical information was sought, and for 4 of these there was no sign of carcinoma for at least 3 years after the high value.

The results are given in Table II. All patients in this table, except those dying of colorectal carcinoma, have been considered free of malignancy in later analyses.

TABLE II.—*Patients with no recurrence during follow-up and raised CEA levels*

| Patient category | Number of patients with raised CEA |
|---|---|
| Died of CC | 20 |
| Died, other causes | 3 |
| Persistent high values | 1 |
| Spurious | 16 |
| Final value high | 6 |
| Total | 46 |

*Deaths of those with no recurrence*

Finally, it is necessary to search through the remaining patients (*i.e.* those with no sign of recurrence during their follow-up in the study, and whose plasma CEA levels remained below 40 ng/ml) to ascertain the causes of death for those who died. This is important, as paients may have died suddenly, of causes relating to the colorectal malignancy, after having no indication during the follow-up examinations and no sign *via* a raised plasma CEA value. In fact, of the 51 who died, 26 died of causes directly related to colorectal carcinoma, 3 died of other malignancies not apparent during follow-up, and 22 died of non-malignant causes. Of the 26 dying of colorectal carcinoma, 7 were examined within 6 months of death and a further 6 were seen between 6 months and 1 year before death; no recurrence was noted at these examinations.

### DISCUSSION

Of 468 patients treated by successful surgery for colorectal carcinoma, and clear from signs of malignancy at the first follow-up examination, recurrence was confirmed during their lifetime in 55 (31 + 12 + 12 (all Table I)) and a further 53 (7 (Table I) + 20 (Table II) + 26) died of colorectal malignancy. Of the 55, 31 showed prior warning of the confirmed malignancy *via* a plasma CEA level $\geqslant 40$ ng/ml. Of the 53 who died, 27 had a raised CEA level sometime before death.

TABLE III.—*Recurrence or death from colorectal carcinoma (CC)*

| | 0–3 months | 3–6 months | 6 months–1 year | 1–2 years | > 2 years | Total |
|---|---|---|---|---|---|---|
| No. of patients with first raised CEA and later definite recurrence while alive | 3 | 7 | 9 | 10 | 2 | 31 |
| No. of patients with first raised CEA who later died of CC | 5 | 0 | 8 | 9 | 5 | 27 |

Table III shows the number of these patients against time before recurrence or death when the plasma CEA level was first raised.

The plasma CEA level was first raised >1 year before recurrence in 12 patients, and >1 year before death in 14. It was raised in 2 patients >2 years before a recurrence was detected and in 5 >2 years before death.

A word of caution is called for in examining these data, relating to their nature and in particular to the pooling of information on patients who had different histories and who underwent different follow-up schedules. For example, the patients whose first raised CEA level was within 3 months of the detection of recurrence could comprise patients who:

(a) developed recurrence immediately after the operation (after being clear of signs of recurrence at the first follow-up);

(b) patients with very sporadic follow-ups but seen within 3 months of a recurrence being detected;

(c) patients with regular follow-ups, and whose plasma CEA level remained "normal" until within 3 months of a recurrence being detected.

In fact, 1 patient belonged to category (a) and 2 to category (c). However, it should be noted that once the CEA level rises, in patients with recurrence, it remains high. Once a high CEA level has been noted in a patient, there must always exist the possibility that, had a CEA test been carried out earlier, the level would have been raised then also. This means that any interpretation of data from a study such as this, where follow-up was irregular, to the effect that a CEA test is helpful, must be considered relevant; *i.e.* in such a study, CEA is given less chance to prove itself, and therefore any indication that the CEA test is useful, must be meaningful.

For the total of 468 patients, it is possible to classify each as +ve or −ve to the CEA test (patients with at least 1 raised CEA level being defined as +ve) and also according to whether malignancy developed or not. Table IV shows the resulting 2 × 2 classification. The data over the whole of the time-span on each patient are greatly simplified in this table, and as stated previously there is a different amount of information on each patient. It is worth noting here the scope for misclassification. As mentioned previously, there is the possibility that a malignancy may have been overlooked or had not manifested itself to be detectable at routine follow-up. If the CEA level for such a patient were raised, this would be classified as a false-positive CEA result. Another obvious source of misclassification concerns the 26 patients with always low CEA levels, who died of colorectal carcinoma. Twelve of these patients were not examined (and no plasma sample taken for CEA determination) for >1 year before death and 6 for >2 years before death. For these patients the CEA test is showing a false-negative result, but if further plasma specimens had been taken before death. the picture may have been different.

TABLE IV.—*CEA-test classification and presence of CC*

| | CC + | CC − | Total |
|---|---|---|---|
| CEA test | | | |
| + | 70 | 32 | 102 |
| − | 38 | 328 | 366 |
| Total | 108 | 360 | 468 |

The strong association between a positive CEA result and colorectal malignancy is apparent for each of the 3 Dukes' classifications considered separately (A, B and C).

An estimate of the true positive rate of the CEA test can be made; this is the percentage of patients with malignancy out of those who are positive to the test. (The range in parentheses represents a 95% confidence interval.)

True positive rate =

$$\frac{70}{102} = 69\% \ (59\text{–}78)$$

This provides a guide to the interpretation of a positive CEA result. The largest groups of patients who were positive to the test but did not have malignancy are the "spurious" group, *i.e.* those with a single raised CEA value followed by lower ones. There is some evidence that a high preoperative CEA level may take some time to return to normal, and that odd single raised values may occur up to 6 months after surgery. However, only 7 out of the 16 had a single high value within 6 months of surgery, and the remaining 9 had the spurious high value > 1 year after the operation.

The true negative rate is also calculated:

$$\frac{328}{366} = 90\% \ (86\text{–}93)$$

For completeness the sensitivity and specificity of the test are given.

The sensitivity of a test is the proportion of those with disease who are positive to the test:

$$\frac{70}{108} = 65\% \ (56\text{–}74)$$

The sensitivity of detection of the disease before other evidence of recurrence can also be calculated:

$$\frac{58}{108} = 54\% \ (44\text{–}64)$$

In 54% of patients developing recurrence or dying from their malignancy, there was a raised CEA level some time before that event, in almost half of these (26 out of 58) the plasma CEA level was raised a year or more earlier.

The specificity of a test is the proportion of those without the disease who show a negative reult. An estimate of this can be made:

$$\frac{328}{360} = 91\% \ (88\text{–}94)$$

Of course, all the rates calculated above refer to the use of CEA in the specific clinical context of monitoring after surgery and one clear follow-up examination for colorectal carcinoma, and do not apply to general-population screening for disease.

### CONCLUSIONS

The results from this study suggest that the plasma CEA test is a useful additional test for the detection of recurrence of colorectal carcinoma. The accepted critical value for the laboratory carrying out these assays is 40 ng/ml and using this, 69% of patients with a raised CEA level were shown by other means to have malignancy. There was prior warning of recurrences in 54% of those who relapsed and a lead time > 1 year was evident in 24% of cases. Of the 70 patients in whom recurrence was noted (Table IV) for 54 there is available at least one further CEA level after the first raised one. In 53 of these, the subsequent CEA levels were also raised; in most cases this was striking. The general conclusion is therefore that once the CEA level becomes elevated in patients with recurrence it remains high and often rises to extremely high levels.

The optimal use to which CEA can be put is an important issue. Decreasing the critical value to 30 ng/ml substantially decreases the true-positive rate for the test, as many more additional patients without recurrence would be positive to the test. The use of consecutive raised values seems indicated, but unfortunately the data from this study do not lend themselves to a detailed analysis of this. The often long interval between consecutive follow-ups mean that had this approach been adopted no patient could be designated as positive until the second of 2 raised values. Tumour may be detected by other means at the second examination and this would mean for these patients no prior warning *via* CEA. It has been suggested that 2 consecutive CEA values > 30 ng/ml may be the best way of using this test. Certain observations regarding this approach can be made from this study. It is to be expected that 2–3% of values from "normal" patients would be > 30 ng/ml. Where CEA is raised in patients with recurrent malignancy and a series of values are available, in most cases the CEA rises quickly and reaches very high levels. For example, 39 patients with recurrence have values > 100 ng/ml, and 15 of these reach values > 1000 ng/ml. A value of between 30 and 40 ng/ml could be due to random variation in a patient in the "normal" state, or the beginning of an increase in CEA level due to malignancy. If CEA rises quickly and there is a long period between follow-up examinations, there would not be expected to be many values in the range 30–40 which are due to malignancy. This appears to be the case with these data. Of the 83 patients in Table I, 17 had at least one CEA value of 30–40 at some stage during their follow-up. Eight of these 17 had a plasma level of < 30 at the next follow-up. These 8 values are quite compatible with the number between 30 and 40 ng/ml which would be expected to occur with chance variation in normal patients. In 5 of these 8, a definite recurrence was detected some time later (in 4/5 cases the CEA levels became greatly increased later in follow-up, after returning to below 30 ng/ml). A further study with more frequent testing of CEA level is needed to determine the best way of using serial CEA values.

The "spurious" single raised CEA levels which returned to normal at the next examination and did not appear to be associated with malignancy again indicate the need for further work. If an approach involving two raised values is to be investigated, the period between consecutive readings could be chosen to ensure that CEA is used in an optimal way. Some current work, in fact, uses two consecutive raised values as a definition of raised CEA level, to study the effect of an intervention programme, based on CEA, on the prognosis for the type of patient considered here (Ratcliffe *et al.*, 1979). Other proposed methods of detecting a significant increase in CEA include the use of a nomogram to compare the postoperative level with a subsequent level (Martin *et al.*, 1977) and an approach using the rate of increase of CEA in plasma (Staab *et al.*, 1978).

The existence of false-negative results also needs further investigation. These occur in patients with malignancy whose plasma CEA levels remain < 40 ng/ml. As a general principle, to obtain the maximum amount of information from a study such as this, the protocol should define strictly the set of tests and examinations to be carried out. These should investigate thoroughly the extent and location of any disease. It may be found that further facets of the disease (metastases in a certain site, stage of differentiation of the tumour, or rate of tumour growth) may be associated particularly with increased plasma levels of CEA, and this information would then be very valuable clinically. Some *in vitro* work has suggested that the output of CEA was inversely proportional to the rate of cell division (Ellison *et al.*, 1977).

Various design problems associated with this study have been mentioned where they are relevant to interpreting the findings. The experience gained from the CEA studies in general should provide sound guidelines for any similar project (Tate, 1981).

### REFERENCES

British Medical Journal (1981) Carcinoembryonic antigen: Its role as a marker in the management of cancer. (Summary of an N.I.H. consensus statement.) *Br. Med. J.*, **282**, 373.

Ellison, M. L., Lamb, D., Rivett, J. & Neville, A. M. (1977) Quantitative aspects of carcinoembryonic antigen output by a human lung carcinoma cell line. *J. Natl Cancer Inst.*, **59**, 309.

Laurence, D. J. R., Stevens, U., Bettleheim, R. & 6 others (1972) Evaluation of the role of plasma carcinoembryonic antigen (CEA) in the diagnosis of gastrointestinal, mammary and bronchial carcinoma. *Br. Med. J.*, iii, 605.

Martin, E. W., James, K. K., Hurtubise, P. E., Cataland, P. & Hinton, J. P. (1977) The use of CEA as an early indicator for gastrointestinal tumour recurrence and second-look procedures. *Cancer*, **39**, 440.

Ratcliffe, J. G., Wood, C. B., Burt, R. W., Malcolm, A. J. H. & Blumgart, L. H. (1979) Patterns of change in carcinoembryonic antigen (CEA) levels in patients after "curative" surgery for colorectal cancer. In *Carcino-Embryonic Proteins*, Vol. II (Ed. Lehmann). Amsterdam, Elsevier North-Holland.

Staab, H. J., Anderer, F. A., Stumpf, E. & Fischer, R. (1978) Slope analysis of the postoperative as an aid in diagnosis of disease progression in gastrointestinal cancer. *Am. J. Surg.*, **136**, 322.

Tate, H. (1981) Assessing tumour markers. *Br. J. Cancer*, **44**, 643.